# Study on browning mechanism of fresh-cut eggplant (*Solanum melongena* L.) based on metabolomics, enzymatic assays and gene expression

**DOI:** 10.1038/s41598-021-86311-1

**Published:** 2021-03-25

**Authors:** Xiaohui Liu, Aidong Zhang, Jing Shang, Zongwen Zhu, Ye Li, Xuexia Wu, Dingshi Zha

**Affiliations:** 1 Horticultural Research Institute, Shanghai Academy of Agricultural Sciences, Shanghai, 201403 China; 2 Shanghai Key Laboratory of Protected Horticultural Technology, Shanghai, 201403 China; 3 Shanghai Ocean University, Shanghai, 201306 China; 4 Harbin Academy of Agricultural Sciences, Harbin, 150070 China

**Keywords:** Metabolomics, Physiology, Plant sciences

## Abstract

Enzymatic browning is one of the crucial problems compromising the flavor and texture of fresh-cut fruit and vegetables. In this study, an untargeted metabolomics approach based on liquid chromatography-mass spectrometry (LC–MS) was used to explore the browning mechanism in fresh-cut eggplant. Metabolomics studies showed that with the increase of fresh-cut time, the contents of 946 metabolites changed dynamically. The metabolites having the same trend share common metabolic pathways. As an important browning substrate, the content of chlorogenic acid increased significantly, suggesting that may be more important to fresh-cut eggplant browning; all 119 common differential metabolites in 5 min/CK and 3 min/CK contrastive groups were mapped onto 31 KEGG pathways including phenylpropanol metabolism, glutathione metabolism pathway, et al. In physiological experiments, results showed that the Phenylpropanoid-Metabolism-Related enzymes (PAL, C4H, 4CL) were changed after fresh-cut treatment, the activities of three enzymes increased first and then decreased, and reached the maximum value at 5 min, indicating the accumulation of phenolic substances. At the same time, ROS were accumulated when plant tissue damaged by cutting, the activities of related antioxidant enzymes (SOD, APX and CAT) changed dynamically after oxidative damage. SOD and APX content increased significantly and reached the maximum value at 10 min after cutting, and then showed a downward trend. However, CAT activity increased sharply and reached the maximum value within 3 min after cutting, then maintained the same activity, and showed a downward trend after 30 min. These data fully demonstrated that the activities of browning related enzymes and gene expression increased with the prolonging of fresh cutting time. We explained the browning mechanism of fresh-cut eggplant by combining metabolomics and physiology, which may lay the foundation for better understanding the mechanism of browning during the fruits and vegetables during processing.

## Introduction

*Solanum melongena* L. is commonly known as eggplant, and belongs to genus Solanum, which is one of the largest genera within family Solanaceae with over 1550 species^1^. It is a common vegetable, rich in polyphenols, dietary fiber, vitamins and other nutrients; it provides a variety of health benefits such as lowering blood lipids, protecting the liver, and as an antioxidant, and thus, it is favored by consumers^2^. However, browning of eggplant occurs easily after peeling, which affects its sensory quality and nutritional value, leading to a decline in edibility and reduced value as a commodity. Browning is the result of a series of physiological and biochemical processes in fruits under aging or adverse conditions. Browning reactions can be divided into two categories, namely enzymatic browning and non-enzymatic browning^3^.

Enzymatic browning is a common phenomenon in the processing of fruits and vegetables. Factors involved in browning reactions of fruits and vegetables, such as apple^4^, pear^5^, luffa^6^ and potato^7^, have been extensively. Researchers have found that the primary reason for browning may be the disruption of the membrane system^8^. When fruits and vegetables are damaged mechanically or in an adverse environment, polyphenols inside them will oxidize under the catalytic action of polyphenol oxidase (PPO) and turn brown^9^, and this requires the participation of oxygen, enzymes and substrates. Peroxidase (POD) and lipoxygenase (LOX) are also key enzymes that cause the browning of fruits and vegetables. Phenolic enzymes and phenolic substances in healthy fruits are regionally distributed in cells; they cannot come into contact when the cells are intact, and no browning reaction occurrs^10^. When the cells suffer adversity, the activities of reactive oxygen scavenging enzymes such as superoxide dismutase (SOD), catalase (CAT) and ascorbate peroxidase (APX) are reduced, which decreases the fruit's ability to scavenge free radicals, and thereby allows the accumulation of reactive oxygen species (ROS)^11^. At the same time, the process of membrane lipid peroxidation is accelerated and membrane permeability is increased, and contact between enzymes and substrate will induce enzymatic browning^12^. In addition, studies have shown that transcription factors also have an impact on fruits and vegetables browning, for example, WRKY gene family may also be involved in the regulation of Luffa^6^ and husk of walnut^13^ browning.

Metabolomics is a burgeoning omics technology, and complements the technologies of genomics and proteomics; Fiehn proposed in 2002 that metabolomics is a qualitative and quantitative analysis method for total metabolites in the body of an organism^14^. With the broadening base of plant metabolomics research, we have gained a better understanding of plant growth regulation, development, metabolic processes and molecular mechanisms of stress responses. In addition, plant metabolomics has become a powerful tool for probing physiology and biology all aspects of plants^15^. In order to explore the mechanism of climate and development regulating the antioxidant system and metabolism of date palm leaves, Du et al. used the non-targeted metabolomics method based on LC–MS to find that young leaves may be more responsive to the environmental change^16^. Using targeted metabolomics, Tang et al. found that levels of hyperoside, POD, SOD, H_2_O_2_, ascorbic acid (AsA), and expression of nine genes (*MdPAL*, *MdCHS*, *MdCHI*, *MdANS*, *MdFLS*, *MdANR*, *MdUGT71K1s*, *MdTTG1* and *MdMYB1*) were lower levels in browning-sensitive apples^4^.

This study focuses on mechanisms behind the browning responsive of ‘Huqie 5′ fresh-cut eggplant fruits by combining plant physiological and metabolomics approaches. The primary purpose of this study was to clarify the biochemical and molecular mechanism of the browning process in fresh-cut eggplant fruits, offer a feasible theoretical foundation for the processing and production of fresh-cut fruit and vegetable, and provide a reference for further investigation into means for reducing the browning of other fresh-cut products.

## Results

### Multivariate analysis

**Figure 1:**
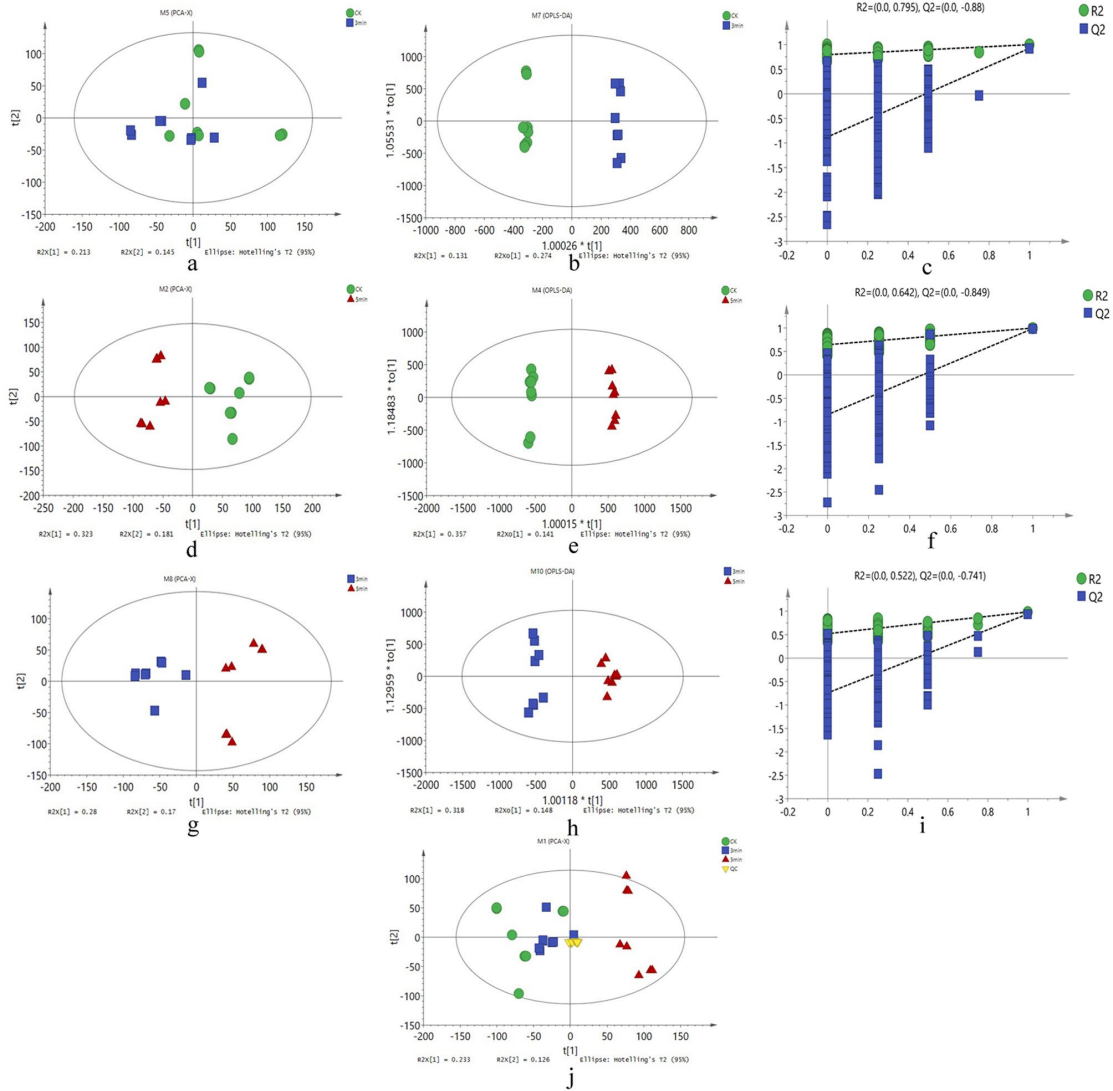
Score scatter plots from PCA, PLS-DA, OPLS-DA and validation plots of OPLS-DA for three comparative groups. (**a**) PLS-DA for 3 min/CK comparative group. (**b**) OPLS-DA for 3 min/CK comparative group. (**c**) Validation plots of OPLS-DA for 3 min/CK comparative group. (**d**) PLS-DA for 5 min/CK comparative group. (**e**) OPLS-DA for 5 min/CK comparative group. (**f**) Validation plots of OPLS-DA for 5 min/CK comparative group. (**g**) PLS-DA for 3 min/5 min comparative group. (**h**) OPLS-DA for 3 min/5 min comparative group. (**i**) Validation plots of OPLS-DA for 3 min/5 min comparative group. (**j)** Score scatter plots of PCA for 3 min/CK comparative group, 5 min/CK comparative group and 3 min/5 min comparative group.

In the data matrix, the data were subject to multivariate analysis tools (Principal Components Analysis (PCA), Partial Least Squares Discrimination Analysis (PLS-DA) and Orthogonal projections to latent structures Discrimination Analysis (OPLS-DA)). PCA, PLS-DA and OPLS-DA scoring plots and validation plots of the OPLS-DA models were built for the three contrastive groups: 3 min/CK, 5 min/CK and 3 min/5 min (Fig. 1). Table 1 displays all the parameters of these models. In our results, all values for Hotelling’s T2 were 95%, Q2 were < 0 (Q2 =  − 0.849 to − 0.741), R2X were > 0.4, and R2 were > 0.5, indicating that the model is reliable.

**Table 1:** The parameters for the assessment of these models.

No. Model		Type	A	N	R2X(cum)	R2Y(cum)	Q2(cum)	R2	Q2
All	M1	PCA-X	6	32	0.651		0.356		
5 min/CK	M2	PCA-X	3	16	0.62		0.416		
5 min/CK	M3	PLS-DA	2	16	0.502	0.989	0.955		
5 min/CK	M4	OPLS-DA	1 + 2 + 0	16	0.64	0.998	0.987	0.642	−0.849
3 min/CK	M5	PCA-X	4	16	0.612		0.106		
3 min/CK	M6	PLS-DA	3	16	0.479	0.995	0.889		
3 min/CK	M7	OPLS-DA	1 + 3 + 0	16	0.595	0.998	0.925	0.795	− 0.88
3 min/5 min	M8	PCA-X	4	16	0.662		0.321		
3 min/5 min	M9	PLS-DA	3	16	0.562	0.995	0.956		
3 min/5 min	M10	OPLS-DA	1 + 1 + 0	16	0.466	0.985	0.943	0.522	− 0.741

*Note* A represents the number of principal components (PC) while each model is constructed, N represents the numbers of samples analyzed, M1-M10 represents Model 1–10, R2X (cum) represents the interpretation rate of each model in the X axis direction in multivariate statistical analysis modeling, R2Y (cum) represents the interpretation rate of each model in Y axis direction in multivariate statistical analysis modeling, Q2 (cum) represents the prediction rate of each model, R2 represents the intercept value of the Y axis and the regression line, which is obtained when Linear regression analysis between the Y matrix of the original classification, the Y matrices of N times’ different permutations and R2Y was conducted during model validation, and Q2 for the intercept value of the Y axis and the regression line, which is obtained when Linear regression analysis between the Y matrix of the original classification, the Y matrices of N times’ different permutations and Q2Y was conducted during model validation. For Q2 in external validation, general requirement is that Q2 < 0, overfitting is avoided. For R2X, general requirement is that R2X > 0.4, the model is good. For R2 in internal validation, general requirement is that R2 > 0.5, the closer to 1, the better the model.

### Differential metabolites

**Figure 2:**
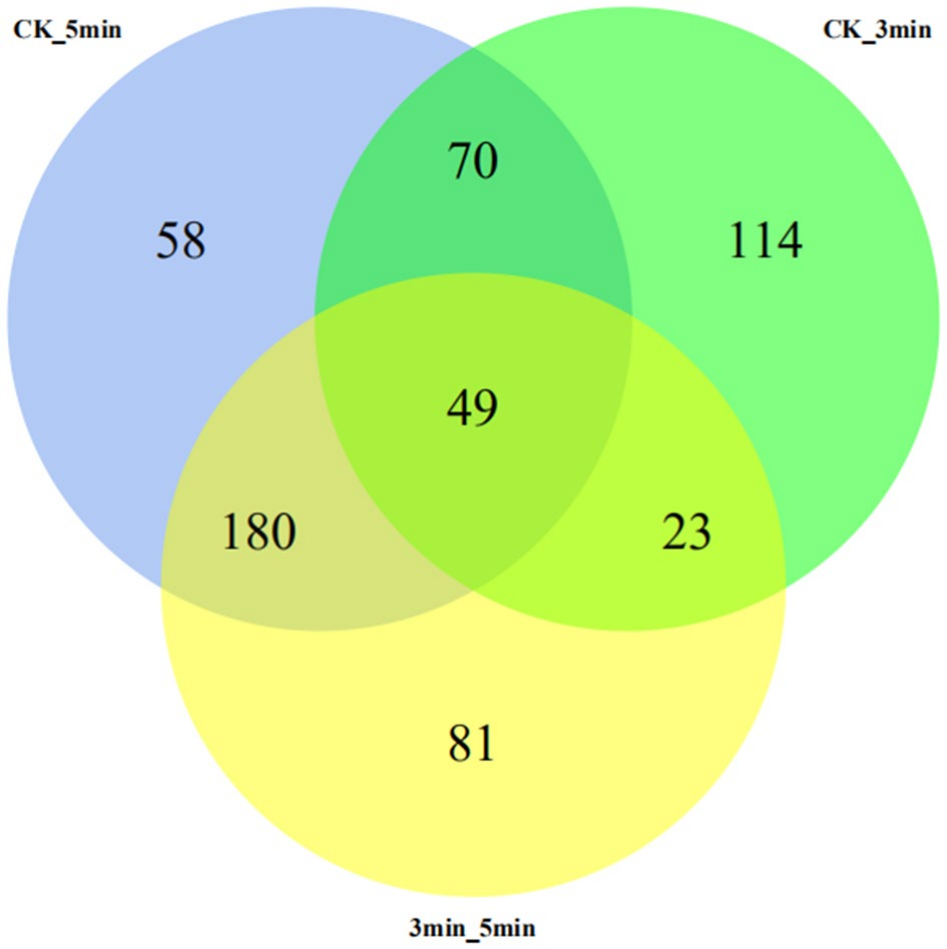
Venn diagram of the differential metabolites among three comparative groups.

Criteria for screening differential metabolites were variable influence of projection (VIP) > 1 for the first principal component in the OPLS-DA, and p-value < 0 (Table S4). Among these differential metabolites, chlorogenic acid is a differential metabolite in 5 min/CK (Fold Change, FC = 1.64) and 5 min/3 min (FC = 1.63) (Table S4). 256 differential metabolites were detected in the 3 min/CK comparative group, 357 in the 5 min/CK comparative group and 333 in the 3 min/5 min comparative group. Among these metabolites, 49 were common to all three groups and 119 were common between 3 min/CK and 5 min/CK. However, 253 differential metabolites were the non-repeated between any two groups; 114, 58 and 81 metabolites were exclusive to the 3 min/CK, 5 min/CK and 3 min/5 min comparative groups, respectively (Fig. 2).

### Heatmap of 119 common differential metabolites

**Figure 3:**
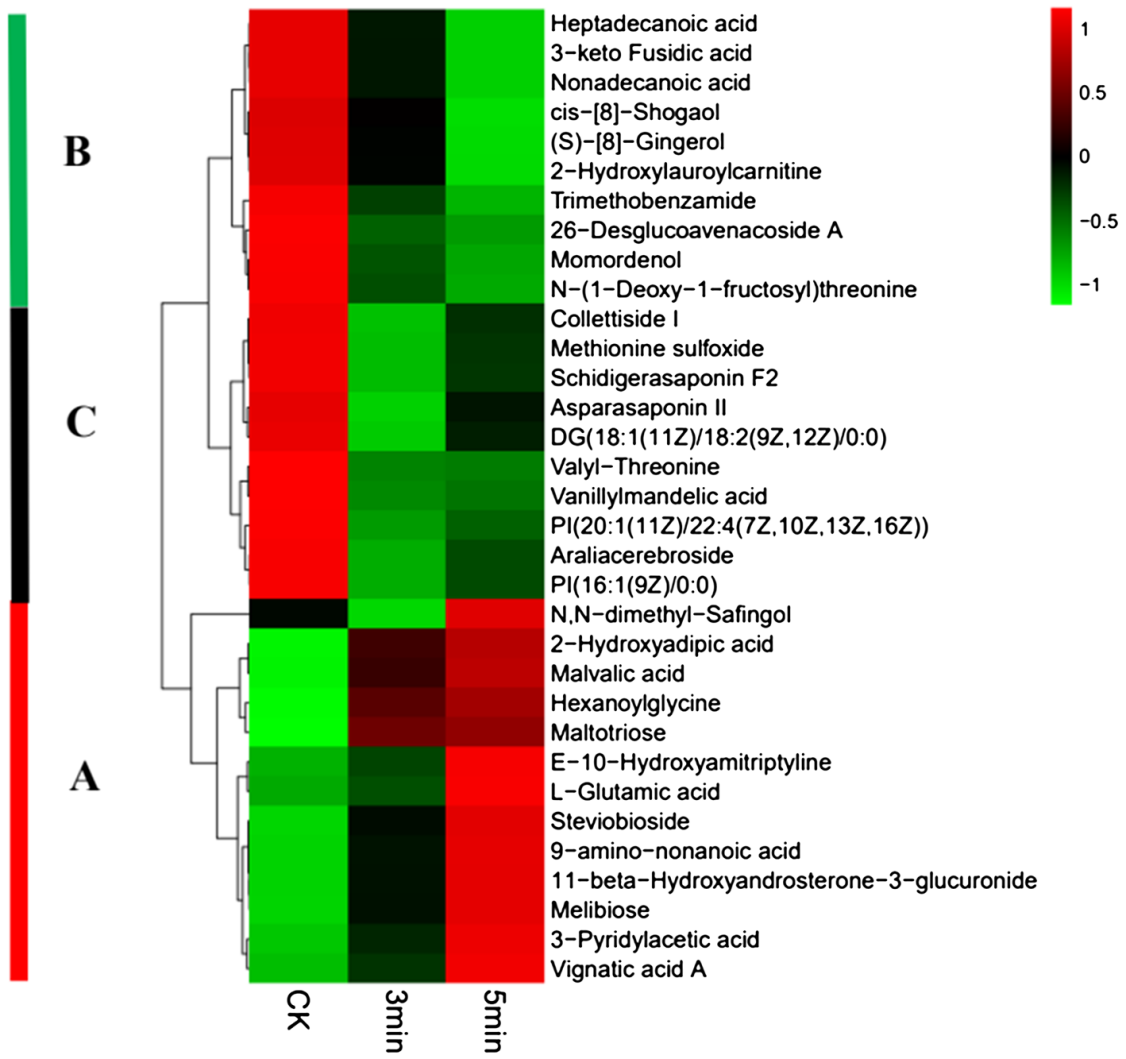
Heatmap showing the differences in expression levels of metabolites.

The 119 common differential metabolites from the 3 min/CK and 5 min/CK groups were further divided into three categories based on STC (Subject To Classification), and are represented by A, B and C in Table S5. Furthermore, the metabolites in these three categories were grouped into eight types of compounds (Table 2). Category A included 68 metabolites, which were mainly lipids, fatty acid and carbohydrates (Table 2). The contents of these metabolites increased gradually. The metabolites of category B comprised 40 compounds, and were mostly fatty acids and lipids (Table 2). The contents of these metabolites decreased as the time from cutting increased. There were 11 components in category C, mainly lipids (Table 2). Interestingly, their contents and expression levels decreased at 3 min, but increased at 5 min. However, they were lower than those at 0 min (CK) as a whole. In order to express the clustering relationships between A, B and C, the peak areas of 33 metabolites were selected to construct a heatmap (Fig. 3).

**Table 2:** Classification of metabolites.

	Benzenoids	Fatty acids	Lipids	Organic acids	Organoheterocyclic compounds	Carbohydrates	Amines	Hydrocarbons
A	1	13	22	6	9	17	0	0
B	3	14	13	3	4	1	1	1
C	1	1	7	2	0	0	0	0

### Metabolite content changes

**Figure 4:**
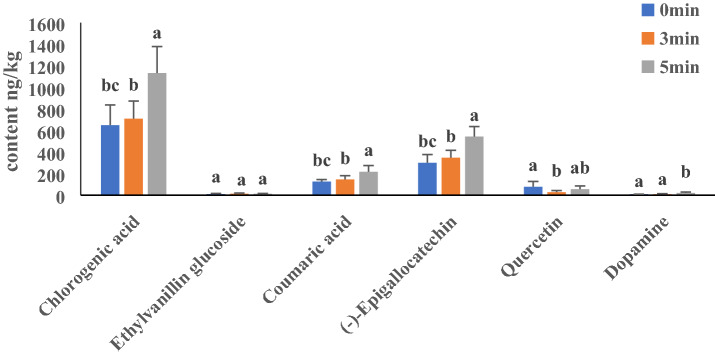
Contents of phenolic substances related to browning.

Six phenols associated with browning were selected from the data matrix. The content levels of the selected metabolites at different treatment intervals are shown in Fig. 4. Fresh-cut treatment promoted the continuous increase of the contents of chlorogenic acid, (−)-epigallocatechin, and coumaric acid, and there was an increase of 42.7%, 41.5% and 44.6% from min 0 to min 5 after cutting, respectively (Fig. 4). However, there was no remarkable alteration in the content of some metabolites, such as ethylvanillin glucoside, quercetin, and dopamine (Fig. 4). These results show that polyphenols are the basic substances for browning. The content of chlorogenic acid was highest at 5 min after cutting, suggesting that it may be more important to browning activity in fresh-cut eggplant.

### KEGG pathway analysis

**Figure 5:**
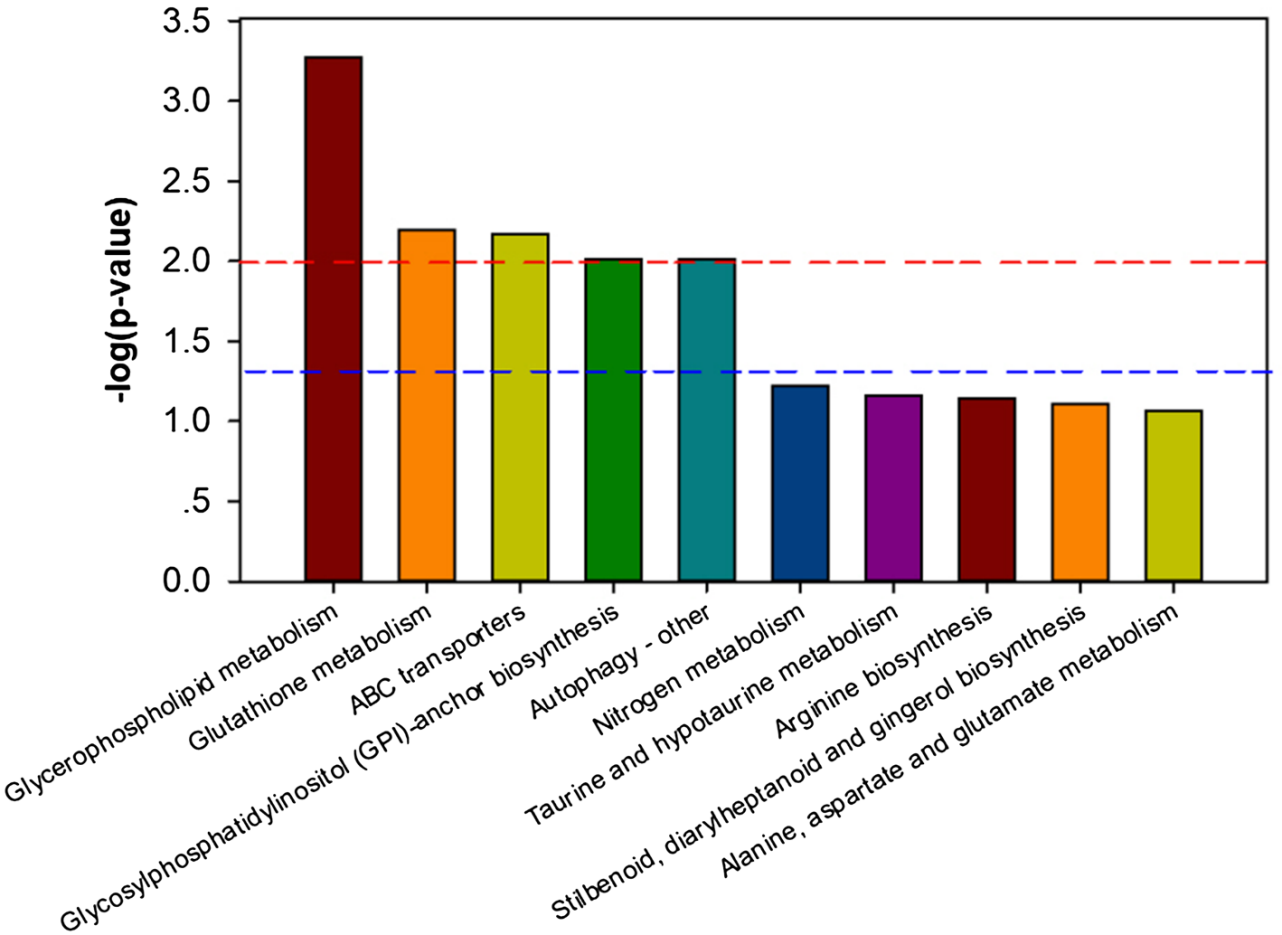
KEGG enrichment of the differential metabolic pathways.

Subsequently, all 119 common differential metabolites in 5 min/CK and 3 min/CK contrastive groups were annotated to biological pathways in the KEGG database using MetaboAnylst 3.0 (Table S6), and the top-10 KEGG pathways are shown in Fig. 5; these include glycerophospholipid metabolism, glutathione metabolism, ABC transporters, glycosylphosphatidylinositol (GPI)-anchor biosynthesis, nitrogen metabolism, taurine and hypotaurine metabolism, arginine biosynthesis, stilbenoid, diarylheptanoid and gingerol biosynthesis, alanine, aspartate and glutamate metabolism. Moreover, phenolic metabolic pathways such as phenylpropanoid biosynthesis and flavonoid biosynthesis were also enriched.

### ROS contents of fresh-cut eggplants

**Figure 6:**
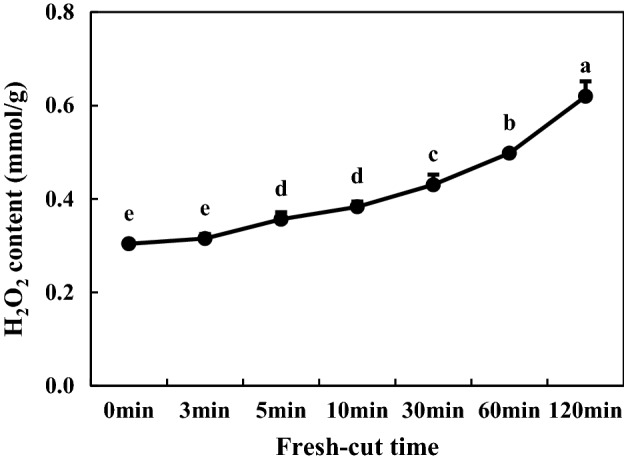
H_2_O_2_ content of Fresh-Cut eggplants.

**Figure 7:**
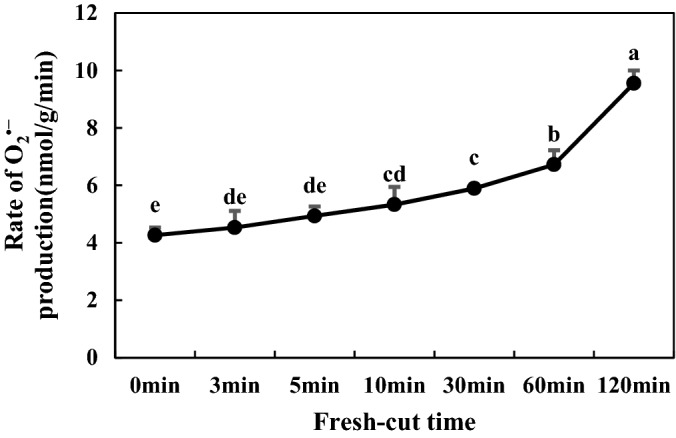
Rate of O_2_**·**− production of Fresh-Cut eggplants.

H_2_O_2_ contents and the rate of O_2_**·**− production in fresh-cut eggplants were also determined for investigation of ROS levels in fresh-cut eggplants. As shown in Fig. 6, H_2_O_2_ content exhibited a slow but steady increasing trend within 0–30 min after fresh-cut treatment. However, the accumulation of H_2_O_2_ from 30 to 120 min showed a significant growth trend (p < 0.05). Compared with CK, the H_2_O_2_ content increased by 50.9% at 120 min after cutting. In addition, a similar tendency was also found in the rate of O_2_**·**− production (Fig. 7). Between 60 and 120 min after cutting, the rate of O_2_**·**− production showed a sharp increase of 29.6%.

### MDA contents of fresh-cut eggplants

**Figure 8:**
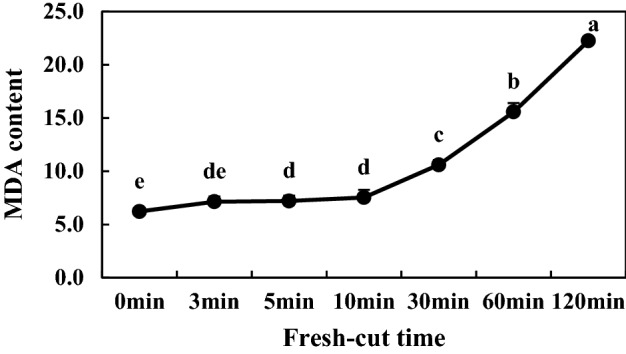
MDA content of Fresh-Cut eggplants.

Malondialdehyde **(**MDA) is one of the products of membrane lipid peroxidation. The content of MDA can represent the damage of the membrane system of fruits and vegetables, and is an important index to evaluate the decomposition of fresh-cut products. The degree of cell membrane lipid peroxidation caused by cutting increases with time along with MDA content of fresh-cut eggplant. It can be seen from Fig. 8 that MDA content always presents an increasing trend during the storage period of freshly cut eggplant. During the whole process, MDA was the highest at 120 min after fresh cutting treatment, indicating that the degree of membrane lipid peroxidation was high and the browning of fresh-cut eggplant was serious.

### Activities of PPO, POD, and LOX and relevant gene expression

**Figure 9:**
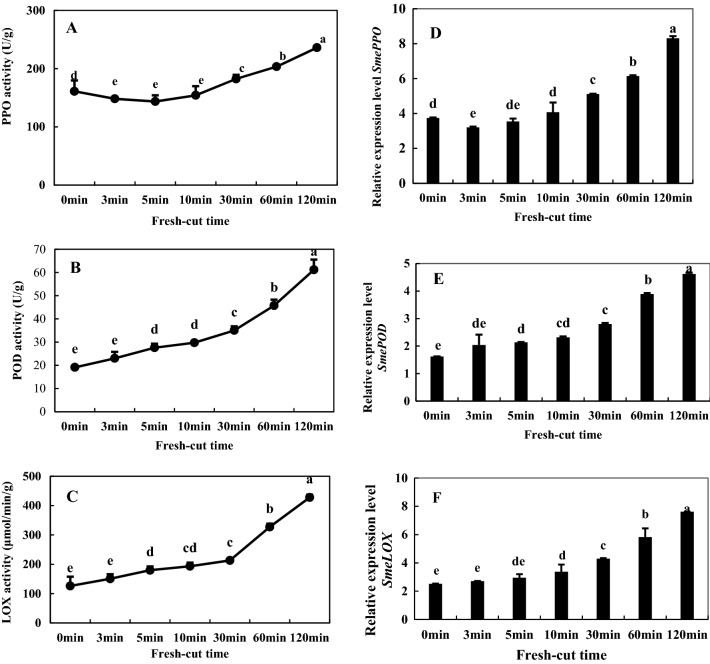
Activities of polyphenol oxidase (PPO) (**A**), peroxidase (POD) (**B**), and lipoxygenase (LOX) (**C**) and gene expression levels of *SmePPO* (**D**), *SmePOD* (**E**), and *SmeLOX* (**F**) of fresh-cut eggplant. Data represent the means of three replicates and their standard errors.

It can be seen from Fig. 9, A, B, C that the activity of PPO, POD and LOX increased with time after fresh cutting; compared with 0 min, they showed increases of 31.7%, 68.7% and 70.5% at 120 min, respectively. Figure 9D–F shows the changes of expression levels of genes encoding PPO, POD, and LOX relative to time after cutting, respectively. The changes in expression levels of *SmePPO*, *SmePOD*, and *SmeLOX* gene were consistent with activity changes of the respective enzymes.

### Activities of PAL, C4H, and 4CL and relevant gene expression

**Figure 10:**
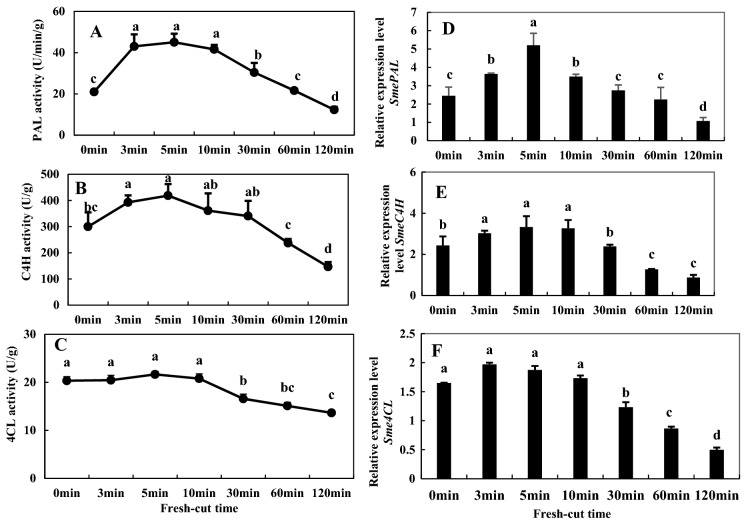
Activities of phenylalanine ammonium lyase (PAL) (**A**), cinnamate-4-hydroxylase (C4H) (**B**), and 4-coumarate coenzyme A ligase (4CL) (**C**) and the gene expression levels of *SmePAL* (**D**), *SmeC4H* (**E**), and *Sme4CL* (**F**) of fresh-cut eggplant. Data represent the means of three replicates and their standard errors.

The changes in activities of phenylalanine ammonia-lyase (PAL), cinnamate 4-hydroxylase (C4H), and 4-coumaric acid: coenzyme A ligase (4CL) relative to time after fresh cutting of eggplant are shown in Fig. 10A–C. The activity of PAL increased significantly in the initial stage (0–5 min) of fresh-cut treatment, peaked at 5 min, and then decreased gradually. A similar tendency was also found in the activity of C4H, which increased sharply during the first 5 min, and was followed by a gradual decrease in the subsequent time. The activity of 4CL showed virtually no significant change during the initial stage (0–5 min), and declined very slowly thereafter, resulting in a 32.9% decrease at the last stage (120 min) compared to 0 min. Figure 10D–F shows the changes in expression levels of *SmePAL*, *SmeC4H*, and *Sme4CL*, respectively; these were consistent with the changes in activities of the respective enzymes.

### Activities of SOD, APX, and CAT and relevant gene expression

**Figure 11:**
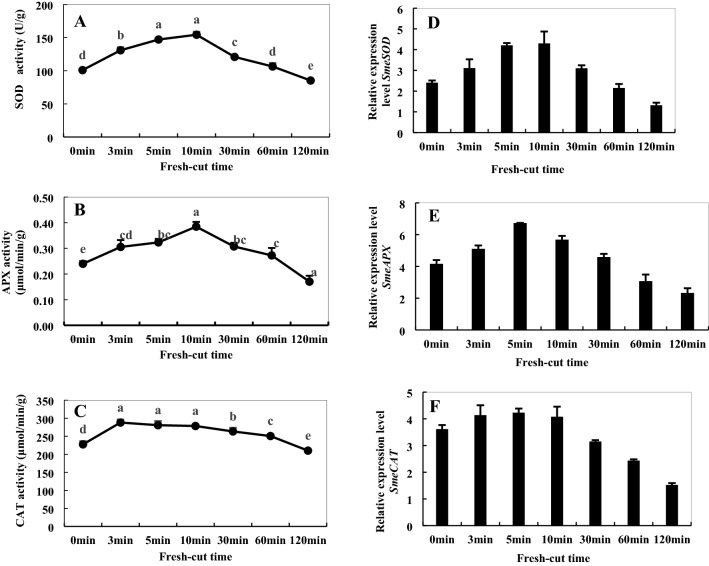
Activities of superoxide dismutase (SOD) (**A**), ascorbate peroxidase (APX) (**B**), and catalase (CAT) (**C**) and gene expression levels of *SmeCu/ZnSOD* (**D**), *SmeAPX* (**E**), and *SmeCAT* (**F**) of fresh-cut eggplant. Data represent the means of three replicates and their standard errors.

As shown in Fig. 11A–C, the activity of SOD increased significantly in the initial stage (0–10 min) of fresh-cut treatment, peaked at 10 min, and then decreased gradually. Relative to 0 min, it increased by 34.6% by the final stage. A similar tendency was also found in the activity of APX. The activity of CAT peaked at 3 min after fresh cutting, and then decreased slowly and steadily. Figure 11D–F show the changes of relative expression levels of genes encoding antioxidant enzymes. The expression changes of *SmeSOD*, *SmeCAT*, and *SmeAPX* gene were consistent with the changes in activities of the respective enzymes; however, the transcriptional peak of *SmeAPX* gene was detected at 5 min, which indicated that the enhancement of enzyme activities lagged behind the gene up-regulation.

## Discussion

Phenolic compounds, as the substrate of browning reaction, are important factors causing browning of fruits^17^. After enzymatic browning of fruits, phenols are oxidized to quinones. As one of the substances affecting enzymatic browning, the type and content of phenolic compounds are of great significance to browning characteristics^18^. Liu et al. studied the reaction mechanism of enzymatic browning during potato processing, and analyzed the correlation between substrates and browning^7^. Their results showed that phenolic compounds are closely related to browning, and chlorogenic acid is an important substrate leading to potato enzymatic browning. The phenolic compounds identified in banana peels in recent years were mainly dopamine, followed by chlorogenic acid and coumarin, with dopaminerase oxidation being the main factor for banana browning^19^. Chlorogenic acid and epicatechin are the phenolic compounds with the highest contents in apple fruits^20^, while the substrates in mango are mainly coffeic acid and ferulic acid^21^. In addition, some studies have shown that apple browning index (BI) is negatively correlated with hyperoside^4^. The substrates for litchi peel browning belong to the collated catechenol compounds, which can rapidly interact with PPO to form browning products^11^. Free polyphenols are the main substrates in the browning reaction of longan peels, and the effect of anthocyanins in the browning reaction is not obvious^22^. The contents of polyphenols reflect the antioxidant capacity of fruits and vegetables. Research results of Liu et al. showed that peach phenols consist of chlorogenic acid, neo-chlorogenic acid and protocatechuic acid^23^. Mishra et al. also showed that browning of eggplant is related to increased contents of polyphenols^24^. In this study, six polyphenols, including chlorogenic acid, (−)-epigallocatechin and coumaric acid, ethylvanillin glucoside, quercetin and dopamine were detected in the fresh-cut eggplant fruits. Interestingly, the content of chlorogenic acid was highest at 5 min after fresh-cut, suggesting that may be more important to fresh-cut eggplant browning activity (Fig. 4).

In addition, PAL, C4H, and 4CL play momentous roles in regulating the production of phenolic substances in phenylpropanoid metabolism^25^. l-Phenylalanine rooted in the Shikimate pathway generates trans-cinnamic acid via PAL ammonia solution reaction. Then trans-cinnamic acid enters into phenylpropanol metabolism, and through the catalytic action of C4H and 4CL, coumaric acid, ferulic acid, erucic acid and other intermediates are generated. These intermediates can be further converted into coumarin and chlorogenic acid, and also can form CoA esters. Finally, these are converted to flavonoids, lignin, alkaloids and other secondary metabolites^26^. Tang et al. found a difference of enzymatic browning unrelated to in Fuji apples. Their results showed that during browning, PPO activities and its gene expression, and total phenol content were not correlated with different browning degrees of apples. However, levels POD, SOD, PAL and their gene expression were higher levels in browning- resistant apples^4^. In this study, we detected a phenylpropanol metabolic pathway with accumulation of chlorogenic acid, (−)-epigallocatechin, and coumaric acid. At the same time, the physiological experiments showed that the activities of PAL, C4H, 4CL were increased; these increases were mediated by increases in gene expression levels of *SmePAL*, *SmeC4H* and *Sme4CL* within 0–5 min of fresh cutting*,* and they coincided with increased accumulation of phenolic substances (Fig. 10).

Existing research demonstrates that phenols usually accumulate in vacuoles, and PPO mainly exists in the cytoplasmic membrane and cell walls^27^. Fruit tissue structure and regionalization of the cell space are damaged by fresh cutting, destroying partitions between PPO and substrate polyphenols and allowing contact between enzymes and substrates for catalysis by PPO, and catalyzing the conversion of phenolic compounds into quinone compounds^28^; quinone compounds can further produce enzymatic browning by polymerizing with intracellular macromolecules to deposit pigment on the surface of damaged tissues^29^. In this study, we detected activity and gene expression of PPO, and the results showed that the activity of PPO in fruits and vegetables would generally increase by cutting (Fig. 9A,D). With increased time after fresh cutting, the activity of PPO in eggplant was gradually enhanced, rising from an initial 161.1 U g^−1^·min^−1^ to 236.0 U·g^−1^ min^−1^ at 120 min after fresh cutting (Fig. 9A). In addition, some researchers believe that the browning reaction is at least partly due to the action of POD on polyphenols^30^. It was found that one POD gene was differentially regulated in fresh-cut luffa fruits^31^. In a study on browning of fresh-cut apples, the activity of POD increased with time from fresh cutting. Our results showed that the activity and gene expression of POD increased with time from the fresh cutting of eggplant fruits (Fig. 9B,E), which is consistent with these previous results.

Substrates (phenolic substances), enzymes and reactive oxygen species (ROS) are three material conditions for enzymatic browning. Under normal circumstances, the generation and clearance of ROS is in dynamic equilibrium, and fresh-cut processing leads to an imbalance in ROS metabolism. Excessive generation of free radicals initiates membrane lipid peroxidation and destruction of membrane structure, prompting contact of phenoloxidase with phenolics; in the presence of oxygen, phenolics are oxidized to quinone, resulting in enzymatic browning^32^. In fresh-cut apple, wounds acquired during cutting and storage induce the accumulation of ROS, such as O_2_**·**− and H_2_O_2_^4^. MDA is the main product of membrane lipid peroxidation, and its accumulation reflects the degree of membrane lipid peroxidation and cellular membrane integrity. Its accumulation can cause further damage to cell membranes and accelerate the browning degree of fruits and vegetables^5^. Our studies confirmed that H_2_O_2,_ MDA content and the rate of O_2_**·**− production increased with time after fresh cutting. LOX is an enzyme that uses polyunsaturated fatty acids as catalytic substrates; it can initiate membrane lipid peroxidation, destroy membrane structure, and its activity increases during browning^5^. Similar to a previous study, the enzyme activity and gene expression of LOX increased with time following fresh cutting in our experiment (Fig. 9C,F), suggesting that LOX may play an important role in the degradation of cellular membranes during fresh-cut eggplant browning.

In ROS metabolic pathway, SOD, APX, and CAT are three antioxidant enzymes that regulate the contents of ROS, which protects plants from oxidative stress^33^. SOD can catalyze O_2_**·**− in cells to produce H_2_O_2_ and O_2_. APX and CAT can clear H_2_O_2_. They can clear the ROS produced during metabolic processes, reduce the content of ROS by enzymatic browning reaction, and have an inhibitory effect on browning^34^. In this study, the activities of these three enzymes increased significantly in the initial stage of fresh-cut treatment, peaked at 5/10 min, and then decreased gradually (Fig. 11). The expression changes of *SmeSOD*, *SmeCAT*, and *SmeAPX* gene were consistent with the change in activities of enzymes; however, the transcriptional peak of *SmeAPX* gene were detected at 5 min, which indicated that the enhancement of enzyme activities lagged behind gene up-regulation (Fig. 11B,E). These results suggest that maintaining higher SOD, CAT, and APX may be crucial to browning resistance. At the same time, the glutathione metabolism pathway was found in our metabolome data, and the content of l-Glutamate involved in this pathway increased. APX is a key enzyme in the glutathione cycle, which can effectively remove ROS and improve the antioxidant capacity of plants through this cycle. Generation of ROS and transcription of most of the antioxidant genes increased during the late stage of fresh-cut eggplant browning in this study; these results indicate that changes in the redox state maybe one of the main reasons for fresh-cut eggplant browning.

## Conclusion

This study provides insights into the physiological and molecular mechanisms of fresh-cut eggplant browning by combining plant physiological and metabolomics approaches. The relationship between the gene expression of ROS metabolism, oxidation and reduction reactions, membrane lipid metabolism and enzymatic browning and core physiology was studied. Antioxidant enzymes play an important role in the browning process, and highly active APX can help delay the browning of fresh-cut eggplant. Our results provide a reference for further investigation into mitigation of browning in other fresh-cut products.

## Material and methods

### Plant material and fresh-cut treatments

Eggplant cultivar ‘Huqie 5′ is a breeding line produced by our lab at the Shanghai Academy of Agricultural Sciences, Shanghai, China. This cultivar is a new combination of early maturity eggplant materials with high yield and good quality; it was bred by using excellent germplasm materials and the multi-generation self-crossing separation technique. The growth characteristics of this cultivar are: strong plant growth, long and thin fruit, deep pericarp coloration, strong luster, good quality, high fruiting rate of high-quality commodity, strong propensity for sustained fruiting. The eggplant fruit were used for untargeted metabolomics analyses at 0, 3 and 5 min after. For the molecular experiments, samples were taken at 0, 3, 5, 10, 30, 60, 120 min after peeling. Samples were collected by slicing them into small segments in liquid nitrogen and then storing the slices in a freezer at − 80 °C until analysis.

### Instruments and chemicals

Vortex oscillator (TYXH-I, Shanghai Hanno Instrument Co., Ltd); Automatic rapid sample grinding machine (JXFSTPRP-24/32, Shanghai Jingxin Industrial Development Co., Ltd); Table top high-speed refrigerated centrifuge (TGL-16MS, Shanghai Lu Xiangyi Centrifuge Instrument Co., Ltd); Ultrasonic cleaning machine (SB-5200DT, Ningbo Xinzhi Biotechnology Co., Ltd); Ultra high performance liquid chromatography (ACQUITY UPLC, Waters); Chromatographic column (ACQUITY UPLC BEH C18 (100 mm × 2.1 mm, 1.7 µm), Waters) ; High-resolution mass spectrometry (AB Triple TOF 5600, AB Sciex). We selected methanol, formic acid, acetonitrile and water from CNW Technologies GmbH and l-2-chlorophenylalanine from Shanghai Hengchuang Biotechnology co., LTD. Note: all chemical reagents and solvents were analytical purity or chromatographic grade.

### Metabolomic analysis

#### Sample preparation

Preparation of samples for metabolites extraction was conducted by transferring 80 mg of lyophilized sample to a 1.5 mL Ep tube. The internal standard was 2-chlorine-l-phenylalanine (20 µL, 0.3 mg mL^−1^ soluble in methanol). Each sample was added with a methanol and water mixture (7:3,v/v, 1000 µL) and placed at − 20 °C for 2 min. Samples were ground at 60 Hz for 2 min, followed by 4 °C ultrasound for 30 min after being subject to a brief eddy current. Samples were then placed at − 20 °C for 20 min and were centrifuged at 1000 × g and 4 °C for 15 min. The supernatants (200 µL per tube) were gathered with a crystal syringe, filtered through a 0.22 µm microfilter and transferred to an automatic sampling bottle for LC–MS analysis. The vials were stored at − 80 °C until they were used for LC–MS analysis. QC (Quality control) samples were made from a mixture of each of the samples; the volume of each QC sample was the same as that of the sample. Before use, all extraction reagents were precooled to − 20 °C.

#### LC–MS analysis

The analytical instrument of this experiment is The UHPLC system, which is composed of liquid-mass coupling system consisting of AB Sciex Triple TOF 5600 high resolution mass spectrometer. It was applied to analyze metabolic profiles in both TIC + and TIC- modes.

Chromatographic conditions were as follows: Chromatographic column: ACQUITY UPLC BEH C18 (100 mm × 2.1 mm, 1.7 μm); column temperature: 45 °C; mobile phase: A—water (containing 0.1% formic acid), B—acetonitrile (containing 0.1% formic acid); flow rate: 0.4 mL min^−1^; injection volume: 5 µL. The elution gradient is shown in Table S1. Mass spectrometry conditions are shown in Table S2. In addition, in order to assess the reproducibility of the whole analysis process, we added one QC sample per 8 analysis samples.

#### Data preprocessing and statistical analyses

The original data were disposed using Progenesis QI made by Waters Corporation from Milford of USA. Then, we constructed a data matrix that was inputted into XCMS software (version 14.0, Umetrics, Umeå, Sweden), in which PCA, PLS-DA and OPLS-DA were executed. A confidence interval is a measure of confidence, and usually bigger is better. R2X represents the cumulative interpretation rate of the multivariate statistical analysis modeling, which generally requires R2X > 0.4 for indication that the model is credible. The 95% confidence interval that defines the change of the model is represented by the Hotelling’s T2 region. It is represented as an ellipse in the rating diagram of the model. The reliability of the model is illuminated by the terms R2X or R2Y and Q2. R2X represents the cumulative interpretation rate of the multivariate statistical analysis modeling. R2Y represents the cumulative interpretation rate in the Y-axis model. Therefore, R2X or R2Y indicate the goodness of fit. R2 and Q2 are the parameters of response sequencing test, used to measure whether the model is overfitted, so as to represent predictability, which can be calculated through the cross-validation process. External validation generally requires Q2 < 0 to avoid over-fitting. Internal validation generally requires R2, > 0.5; The closer R2 is to 1, the better the model. To prevent overfitting of the model, seven-fold cross-validation and 200 RPT were applied to evaluate the quality of the model.

#### Identification of differential metabolites

The differential metabolites between groups were screened out using the methods of multi-dimensional analysis and one-dimensional analysis. The screening criteria were VIP > 1 for the first principal component of the OPLS-DA model, and p < 0.05 for the Student’s t-test, where the variation multiple was the ratio of the average metabolites in the two groups. Metabolites were described using Progenesis QI (Waters Corporation, Milford, USA) metabolic data software based on public databases (http://www.hmdb.ca/; http://www.lipidmaps.org/) and a self-built database. Furthermore, the identified metabolites were classified to different categories of compounds using the Human Metabolome Database (HMDB, http://www.hmdb.ca/).

#### KEGG pathway analysis

These differential metabolites were mapped onto the KEGG database, and metabolic pathways were analyzed based on KEGG (http://www.genome.jp/KEGG/pathway.html) pathway analysis.

### Physiological analysis

#### Sample preparation

Fresh frozen eggplant samples (0.2 g) were weighed and then homogenized with an extraction liquid volume (1:5, v/v). The homogenate was centrifuged at 12,000×*g* for 10 min at 4 °C, and the resulting supernatant was placed on ice for subsequent determination.

#### Physiological index assay

The following physiological indices were determined using the appropriate kits according to the instruction manual (Suzhou Grace Biotechnology Co., Ltd., Suzhou, China): Total Phenolics Content (TPC), superoxide radicals (O_2_**·**−), H_2_O_2_, MDA, superoxide dismutase (SOD) activity, ascorbate peroxidase (APX) activity, catalase (CAT) activity, PPO, POD, LOX). TPC content was determined by Folin-Ciocalteu method, and the absorbance was measured at 760 nm to calculate the content of TPC; O_2_**·**− content: the readings at 530 nm were recorded and the results were expressed as nanomolar pergram per minute (nmol g^−1^ min^−1^); H_2_O_2_ content: the reading of 415 nm was recorded to calculate the content; MDA content: The difference of absorbance between 532 and 600 nm was used to calculate the content of MDA. SOD activity: the change of absorbance of reaction solution at 450 nm per minute was 0.001 as one enzyme activity unit, and the result was expressed as U mg^−1^ protein; APX activity: 0.001 change of absorbance of reaction solution at 290 nm per minute was taken as one enzyme activity unit, and the result was expressed as U mg^−1^ protein; CAT activity: 0.001 change of absorbance of reaction solution at 240 nm per minute was taken as one enzyme activity unit, and the result was expressed as U mg^−1^ protein; PPO activity: the change of absorbance of reaction solution at 420 nm per minute was 0.001 as one enzyme activity unit, and the result was expressed as U mg^−1^ protein; POD activity: the change of absorbance of reaction solution at 470 nm per minute was 0.001 as 1 enzyme activity unit, and the result was U mg^−1^ protein; LOX activity: the change of absorbance of reaction solution at 234 nm per minute was 0.001 as 1 enzyme activity unit, and the result was U mg^−1^ protein.

The physiological data for PAL, C4H, 4CL were determined with kits provided by Beijing Solarbio Science & Technology co., Ltd., Beijing, China. PAL activity: PAL activity was calculated by measuring the increase rate of absorption value at 290 nm. The 0.1 change in absorbance value at 290 nm per minute for each g of tissue in the reaction system per mL is defined as an enzyme activity unit; C4H activity: The absorbance value at 340 nm was determined for calculation of C4H activity. The amount of enzyme that reduces 1 nmol NADPH per minute per g of tissue in the reaction system is defined as a unit of enzyme activity; 4CL activity: The absorbance value at 333 nm was determined for calculation of 4CL activity. The production of 1 nmol 4-coumaric acid coenzyme A per minute per g of tissue is defined as A unit of enzyme activity.

#### Quantitative real-time PCR (qRT-PCR) and gene expression assays

qRT-PCR was performed to analyze the expression levels of *SmePPO*, *SmePOD*, *SmeLOX*, *Sme PAL*, *SmeC4H*, *Sme4CL*, *SmeSOD*, *SmeCAT*, *SmeAPX* gene. Total RNA was extracted using the Quick RNA isolation Kit (Beijing TsingKe Biotech Co., Ltd., Beijing, China) according to the manufacturer’s protocol. First-strand cDNA was synthesized from 2 μg of total RNA using M-MLV reverse transcriptase (Beijing TsingKe Biotech Co., Ltd., Beijing, China) and Oligo (dT)18 in a 25-μL reaction. Real-time PCR was performed with SYBR Green PCR mix (Takara, Shiga, Japan). *SmPKG* was used as an endogenous control gene for qRT-PCR analyses. Relative expression levels of the target genes were calculated using the 2− ΔΔCt method. The primers used are listed in Table S3.

### Compliance with ethical standards

All conducted experiments complied with the laws of China.

## Supplementary Information

Supplementary Table S1.

Supplementary Table S2.

Supplementary Table S3.

Supplementary Table S4.

Supplementary Table S5.

Supplementary Table S6.
