# Supplementary material for: Study on browning mechanism of fresh-cut eggplant (Solanum melongena L.) based on metabolomics, enzymatic assays and gene expression
Source: Sci Rep. 2021 Mar 25;11:6937. doi: 10.1038/s41598-021-86311-1 (PMC7994816; doi:10.1038/s41598-021-86311-1)
Supplement: Supplementary file 1 — Supplementary Table S1. [file 41598_2021_86311_MOESM1_ESM.docx]

| Time(min) | A% | B% |
| --- | --- | --- |
| 0 | 95 | 5 |
| 2 | 80 | 20 |
| 4 | 75 | 25 |
| 9 | 40 | 60 |
| 14 | 0 | 100 |
| 18 | 0 | 100 |
| 18.1 | 95 | 5 |
| 19.5 | 95 | 5 |

**Table S1 Elution gradient**
