# Supplementary material for: Study on browning mechanism of fresh-cut eggplant (Solanum melongena L.) based on metabolomics, enzymatic assays and gene expression
Source: Sci Rep. 2021 Mar 25;11:6937. doi: 10.1038/s41598-021-86311-1 (PMC7994816; doi:10.1038/s41598-021-86311-1)
Supplement: Supplementary file 2 — Supplementary Table S2. [file 41598_2021_86311_MOESM2_ESM.docx]

| **parameters** | **positive** | **negitive** |
| --- | --- | --- |
| Nebulizer Gas (GS1, PSI) | 40 | 40 |
| Auxiliary Gas (GS2, PSI) | 40 | 40 |
| Curtain Gas (CUR, PSI） | 35 | 35 |
| Ion Source Temperature （℃） | 550 | 550 |
| Ion Spray Voltage (V) | 5500 | 4500 |
| Declustering Potential (DP ,V) | 100 | -100 |
| Mass Scan Range (TOF MS scan) | 70-1000 | 70-1000 |
| Collision Energy （TOF MS scan, eV） | 10 | -10 |
| Mass Scan Range (Product Ion scan) | 50-1000 | 50-1000 |
| Collision Energy (Product Ion scan,eV） | 30 | 30 |
| Interface Heater Temperature (℃) | 550 | 600 |

**Table S2 MS parameters**
